# The ontogeny of Butyrophilin-like (Btnl) 1 and Btnl6 in murine small intestine

**DOI:** 10.1038/srep31524

**Published:** 2016-08-16

**Authors:** Cristina Lebrero-Fernández, Anna Bas-Forsberg

**Affiliations:** 1 Department of Microbiology and Immunology, Institute of Biomedicine, University of Gothenburg, Gothenburg, Sweden

## Abstract

Murine Butyrophilin-like (Btnl) 1 and Btnl6 are primarily restricted to intestinal epithelium where they regulate the function of intraepithelial T lymphocytes. We recently demonstrated that Btnl1 and Btnl6 can form an intra-family heterocomplex and that the Btnl1-Btnl6 complex selectively expands Vγ7Vδ4 TCR IELs. To define the regulation of Btnl expression in the small intestine during ontogeny we examined the presence of Btnl1 and Btnl6 in the small bowel of newborn to 4-week-old mice. Although RNA expression of *Btnl1* and *Btnl6* was detected in the small intestine at day 0, Btnl1 and Btnl6 protein expression was substantially delayed and was not detectable in the intestinal epithelium until the mice reached 2–3 weeks of age. The markedly elevated Btnl protein level at week 3 coincided with a significant increase of γδ TCR IELs, particularly those bearing the Vγ7Vδ4 receptor. This was not dependent on gut microbial colonization as mice housed in germ-free conditions had normal Btnl protein levels. Taken together, our data show that the expression of Btnl1 and Btnl6 is delayed in the murine neonatal gut and that the appearance of the Btnl1 and Btnl6 proteins in the intestinal mucosa associates with the expansion of Vγ7Vδ4 TCR IELs.

Members of the Butyrophilin (Btn) and Btn-like (Btnl) family are recognized as novel immune regulators. The BTN and BTNL proteins are characterized by their structural relatedness to the B7 co-stimulatory molecules, and as with B7, several of human and murine BTN and BTNL family members are reported to dampen or augment αβ and γδ T lymphocyte responses^1^,^2^,^3^,^4^,^5^,^6^,^7^,^8^,^9^,^10^. Moreover, BTN3A1 reportedly mediates the activation of Vγ9Vδ2 T cells by phosphoantigens^11^,^12^,^13^, suggesting that BTN and BTNL proteins not only have the capacity to regulate T cell-mediated immune responses but are additionally involved in phosphoantigen sensing. In addition, some of the BTN and BTNL molecules have been associated with inflammatory disorders and cancer. Thus: polymorphisms in the human *BTNL2* gene have been linked to pulmonary sarcoidosis, ulcerative colitis, rheumatoid arthritis, myositis and prostate cancer^14^,^15^,^16^,^17^,^18^; human BTN3 has been associated with ovarian cancer^9^,^19^,^20^; *Btn2a2* deficiency was recently described to exacerbate experimental autoimmune encephalomyelitis and to potentiate anti-tumor responses^21^; and we recently presented altered expression of human *BTN* and *BTNL* genes in intestinal inflammation and colon cancer^22^. These accumulating data provide growing evidence that members of the Btn and Btnl family play a diverse and essential role in orchestrating the immune system. We previously demonstrated that Btnl1 can regulate intraepithelial lymphocyte (IEL)–epithelial cell interactions in the murine small intestinal mucosa by attenuating the epithelial response to activated IELs^5^. Furthermore, we recently reported that Btnl1, in a heteromeric complex with Btnl6, selectively enhances the expansion of γδ IELs bearing the Vγ7Vδ4 TCR in the absence of exogenous activation^6^. To define the expression of Btnl1 and Btnl6 in the intestine during ontogeny we investigated the presence of Btnl mRNA and protein in the small intestine of day 0–4-week-old mice. Additionally, we examined the expression of γδ TCR IELs in the neonate gut and characterized the usage of Vγ7 and Vδ4 chains during the first weeks of neonatal development.

## Materials and Methods

### Mice

Germ-free (GF) 8–12-week-old C57BL/6 mice and conventional (CV) C57BL/6 mice were housed in the Laboratory of Experimental Biomedicine (EBM), University of Gothenburg (Gothenburg, Sweden). GF mice were maintained in flexible plastic film isolators under a 12-h light cycle and fed autoclaved chow diet (Labdiet) *ad libitum*. GF status was verified regularly by anaerobic culturing in addition to PCR for bacterial 16S rDNA. CV C57BL/6 mice were bred at EBM and were used in the experiments at 0–8 weeks of age. Pregnant mice were identified and monitored daily until delivery. The day of birth was identified as day 0 of life. The pups were kept in the parental cage until day 21 of age and were thereafter weaned from their mother. Protocols were approved by the Gothenburg animal ethics committee (Göteborgs djurförsöksetiska nämnd; permit no. 335–2012), and institutional animal use and care guidelines were followed.

### RNA extraction and cDNA preparation

Murine small intestinal tissue was lysed and homogenized (TissuelyserII, Qiagen, Valencia, CA) and total RNA was isolated using RNeasy^®^ mini kit (Qiagen, Valencia, CA), including DNAse I digestion. RNA concentration was determined spectrophotometrically (NanoDrop ND-1000, Wilmington, DE). SuperScript^TM^ III Reverse Transcriptase kit (Invitrogen^TM^, Life Technologies, Carlsbad, CA) was used for cDNA synthesis, using 1000 ng RNA as template in total reaction volume of 20 μl.

### Quantitative real-time PCR

Expression of *Btnl1* and *Btnl6* complementary DNA (cDNA) was measured by quantitative real-time PCR using GoTaq^®^ qPCR Master Mix, according to the manufacturer’s instructions (Promega, Madison, WI). The qPCR was performed on a LightCycler480 thermal cycler (Roche Diagnostics, Mannheim, Germany). The PCR primers, spanning exon-exon borders to avoid amplification of genomic DNA, were: 5′-tgaccaggagaaatcgaagg-3′ and 5′-caccgagcaggaccaatagt-3′ for *Btnl1,* and 5′-atccttggagatccacagtgaa-3′ and 5′-gggagagaccttgggaaaga-3′ for *Btnl6*. RNA expression was normalized to the expression of *β-actin* (5′-cttctttgcagctccttcgtt-3′ and 5′-aggagtccttctgacccatgc-3′). Each qPCR analysis was duplicated.

### Generation of stably transfected N-terminal FLAG-tagged Btnl6-pMX-IRES-GFP-MODE-K cells

Stably transfected murine intestinal epithelial MODE-K cells were generated, as previously described^5^. Cells were maintained at 37 °C, 5% CO_2_ in Dulbecco’s modified essential medium (DMEM; Gibco^®^, Life Technologies, Carlsbad, CA) plus 10% FCS (PAA Laboratories, Linz, Austria), 100 Ug/ml penicillin, 100 μg/ml streptomycin, 0.292 mg/ml glutamine, and 1 × non-essential amino acids (Gibco^®^, Life Technologies, Carlsbad, CA).

### Isolation of murine small intestinal epithelial cells and intraepithelial lymphocytes

Intestinal epithelial cells (iECs) and intraepithelial lymphocytes from murine small bowel were isolated according to previously described procedures^23^. IELs were recovered at the interface between 80% and 40% Percoll (GE Healthcare Bio-sciences AB, Uppsala, Sweden), and iECs were recovered at the interface between 40% and 20% Percoll.

### Confocal microscopy

Methanol-Carnoy-fixed and paraffin embedded sections were stained with anti-Btnl1 rabbit polyclonal antiserum or pre-immune serum. Sections were incubated with TRITC-conjugated AffiniPure F(ab’)_2_ fragment donkey anti-rabbit IgG (H+L) (Jackson ImmunoResearch, West Grove, PA). Sections were mounted in ProLong^®^ Gold antifade reagent containing 4′,6-diamidino-2-phenylindole (DAPI) (Molecular Probes^®^, Life Technologies, Eugene, OR). Tissue sections were viewed using confocal microscopy (Zeiss LSM700 Inverted) and analyzed with ZEN lite 2011 microscope software (Carl Zeiss, Oberkochen, Germany).

### Western blotting

Small intestine, harvested from GF and CV C57BL/6 mice, was homogenized in cell lysis buffer (50 mM Tris-HCl, pH 8, 150 mM NaCl, 1% Triton X-100) containing complete protease inhibitors cocktail tablets (Roche Diagnostics, Mannheim, Germany). Small intestinal epithelial cells isolated from CV C57BL/6 mice and Btnl6-transfected MODE-K cells were lysed in cell lysis buffer. Cell and tissue lysates were clarified by centrifugation, and protein concentration was measured with BCA Protein Assay Kit (Pierce, Rockford, IL). Twenty micrograms of protein were denatured in reducing sample buffer (NuPAGE LDS 4×; Novex^®^, Life Technologies, Carlsbad, CA) containing 1M DTT (Sigma-Aldrich, St. Louis, MO) and loaded onto a NuPage 4–12% Bis-Tris Gel (Novex^®^, Life Technologies, Carlsbad, CA). Separated proteins were transferred onto nitrocellulose transfer membranes (Merck Millipore, Darmstadt, Germany) that were immunoblotted using anti-FLAG antibody (Sigma-Aldrich, St. Louis, MO), anti-Btnl6 rabbit polyclonal antiserum (Moravian-Biotech, Brno, Czech Republic), rabbit pre-immune serum, or anti-β-actin antibody (Sigma-Aldrich, St. Louis, MO), and detected with HRP-conjugated goat anti-mouse antibody or HRP-conjugated goat anti-rabbit antibody (Jackson ImmunoResearch, West Grove, PA).

### Flow cytometric analysis

Cell-surface antigen expression was analyzed using the following antibodies: anti-Btnl1 rabbit polyclonal antiserum, pre-immune serum, anti-CD45-Alexa Fluor 700 (30-F11; eBioscience, San Diego, CA), anti-CD3ε-FITC (145–2C11; BD Pharmigen^TM^, San Diego, CA), anti-pan TCRγδ-eFluor450 (eBioGL3; eBioscience, San Diego, CA), anti-TCRβ-APC (H57–597; eBioscience, San Diego, CA), anti-TCR Vγ1.1/Cr4-PE (2.11; BioLegend, San Diego, CA), anti-TCR Vδ4-eFluor660 (GL2; eBioscience, San Diego, CA), anti-TCR Vγ7-biotin (kindly provided by Dr Pablo Pereira, Institut Pasteur, Paris, France), 7-aminoactinomycin D (7AAD; Sigma-Aldrich, St. Louis, MO), and LIVE/DEAD^®^ Fixable Red Dead Cell Stain (Molecular Probes^®^, Life Technologies, Eugene, OR). Detection of anti-Btnl1 and anti-TCR Vγ7 was achieved with APC-conjugated AffiniPure F(ab’)_2_ fragment donkey anti-rabbit IgG (H+L) (Jackson ImmunoResearch, West Grove, PA) and streptavidin-APC-Cy^TM^7 (BD Biosciences, San Diego, CA), respectively. Cells were gated on 7AAD or LIVE/DEAD^*®*^ Fixable Red negative cells to exclude non-viable cells, and positive staining for Btnl1 was determined by comparison with pre-immune serum. Cell samples were acquired on LSR II flow cytometer using the DIVA software (BD Biosciences, San Diego, CA), and analysis of data was perfomed using the FlowJo Software version 7.6.5 (Ashland, OR).

### Statistical analysis

Data were generated using GraphPad Prism version 6.04 (San Diego, CA). The unpaired two-tailed t test was used for comparison between two independent groups, while One-Way ANOVA followed by Holm-Sidak´s multiple comparisons test was applied to evaluate differences between three or more groups. Pearson correlation test was performed to determine the correlation between parameters. Differences were considered as statistically significant when p < 0.05 (*P ≤ 0.05, **P ≤ 0.01, ***P ≤ 0.001 and ****P ≤ 0.0001).

## Results

### Appearance of Btnl1 and Btnl6 in the small intestinal epithelium is delayed during ontogeny

**Figure 1:**
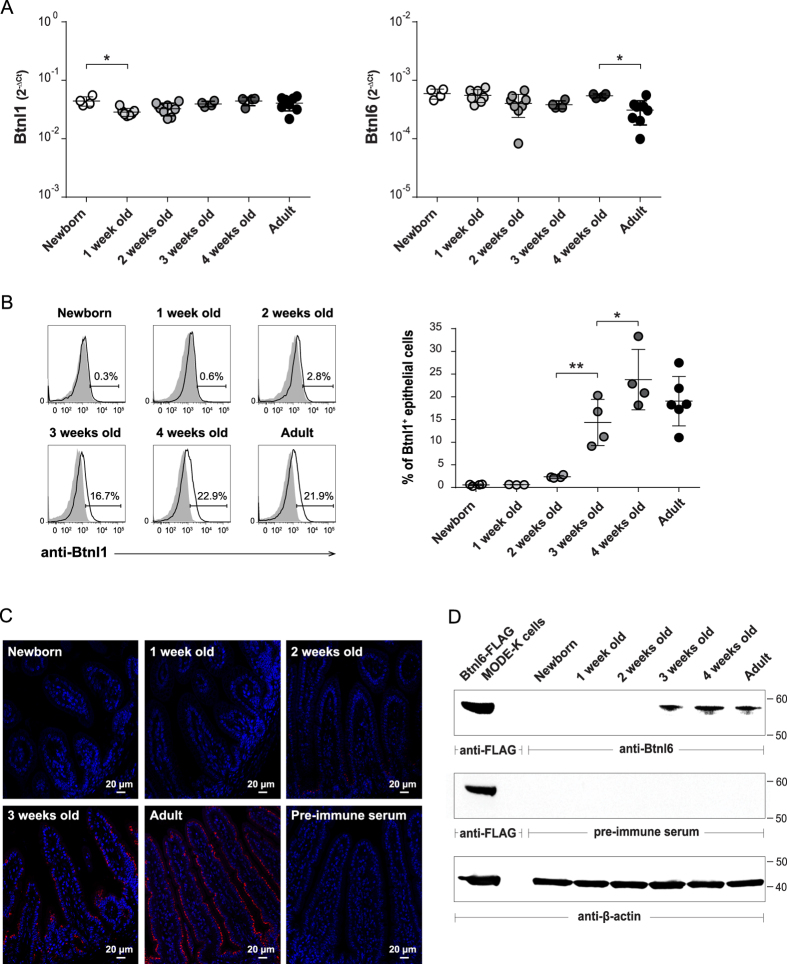
Btnl1 and Btnl6 expression in murine intestine during ontogeny. Expression of *Btnl1* and *Btnl6* genes **(A)**, and Btnl1 **(B,C)** and Btnl6 proteins **(D)** was examined in small intestinal tissue of newborn (day 0), 1, 2, 3, and 4-week-old and adult C57BL/6 mice. **(A)** *Btnl1 and Btnl6* gene expression was assessed by qPCR, run in duplicates, and normalized against β*-actin*. **(B)** Murine intestinal epithelial cells, gated on CD45^−^ epithelial cells and 7AAD negative cells to exclude non-viable cells, were stained with anti-Btnl1 rabbit polyclonal antiserum (solid black line) or pre-immune serum (shaded histogram) which served as a negative control. Representative histogram for each time-point is shown. 4–8 mice/time-point were analyzed and One-Way ANOVA followed by Holm-Sidak´s multiple comparisons test was used for statistical analysis (*P ≤ 0.05, **P ≤ 0.01, ***P ≤ 0.001 and ****P ≤ 0.0001) **(A,B)**. **(C)** Small intestinal sections were immunostained with anti-Btnl1 rabbit polyclonal antiserum (red) and counterstained with DAPI (blue) to visualize nuclei. No staining was detected using pre-immune serum. Original magnification 20x. Four mice were stained for each time-point and representative stainings are shown. **(D)** Isolated iECs from small intestinal tissue of C57BL/6 mice (20 μg) were analyzed for Btnl6 protein expression. Lysates from MODE-K cells transfected with FLAG-tagged Btnl6 cDNA pMX-IRES-GFP served as a positive control. The predicted protein migrating under reducing conditions at the theoretical molecular weight of ~59 kDa for FLAG-tagged Btnl6 and ~58 kDa for non-tagged Btnl6 was detected with anti-FLAG antibody or Btnl6-specific polyclonal antibody. No bands were detected on gels immunoblotted with pre-immune serum. The β-actin immunoblot acts as a loading control. Data are representative of four experiments.

At birth and at weaning, mammals are exposed to multiple novel antigens from diet and microbial colonization. To gain insight into how these events regulate the expression of Btnl1 and Btnl6, small intestinal epithelial cells isolated from newborn mice and from mice pre- and post-weaning were analyzed for Btnl1 and Btnl6 expression. Although *Btnl1* and *Btnl6* transcripts were detected in the small intestine of newborn animals (Fig. 1A), the mRNA expression was not reflected at the protein level by flow cytometry (Fig. 1B), or immunohistochemistry (Fig. 1C), for Btnl1, or by western blot for Btnl6 (Fig. 1D). Directly *ex vivo*, viable 7AAD^−^ CD45^−^ small intestinal cells with side and forward scatter typical of epithelial cells showed increasing levels of surface Btnl1 during intestinal ontogeny; no protein was detected in newborn and 1-week-old pups, the protein appeared when the mice had reached the age of 2 weeks (2.4% ± 0.3) and then increased to reach levels comparable to those detected in adult animals (19% ± 5) by 4 weeks of age (24% ± 7) (Fig. 1B). Whereas flow cytometry analysis detected Btnl1 cell surface expression, the immunostaining of intestinal tissue visualized total protein levels; in 4 μm sections of small intestinal tissue Btnl1 was first detected at 2 weeks of age and, consistent with flow cytometry data, protein expression was found to increase with increasing age (Fig. 1C). As for Btnl1, the expression of Btnl6 proteins was substantially delayed and was not detectable in the intestinal epithelium until 3 weeks of age (Fig. 1D). Thus, the appearance of Btnl proteins occurred in pre-weaning pups kept mainly on a milk diet and was not associated with weaning.

### The delay in Btnl1 and Btnl6 protein appearance in the neonate gut is not associated with gut colonization

**Figure 2:**
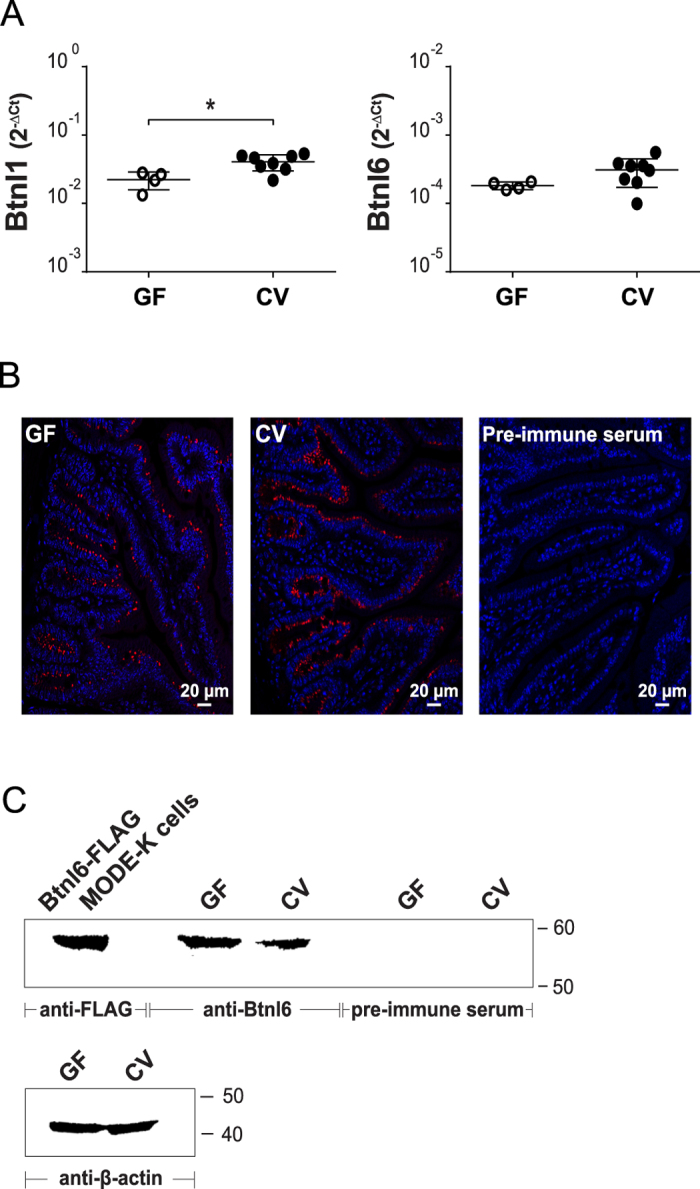
Btnl1 and Btnl6 expression in germ-free mice. Expression of *Btnl1* and *Btnl6* transcripts **(A)**, and Btnl1 **(B)** and Btnl6 proteins **(C)** was analyzed in small intestinal tissue of GF and CV adult C57BL/6 mice. **(A)** *Btnl1* and *Btnl6* gene expression was assessed by qPCR, run in duplicates, and normalized against β*-actin*. 4–8 mice/group were included and unpaired two-tailed t test was used for statistical analysis (*P ≤ 0.05, **P ≤ 0.01, ***P ≤ 0.001 and ****P ≤ 0.0001). **(B)** Small intestinal sections were immunostained with anti-Btnl1 rabbit polyclonal antiserum (red) and counterstained with DAPI (blue) to visualize nuclei. No staining was detected using pre-immune serum. Original magnification 20x. Four mice were stained for each group and representative stainings are shown. **(C)** Lysates from small intestinal tissue of GF and CV adult C57BL/6 mice (20 μg) were analyzed for Btnl6 protein expression. Lysates from MODE-K cells transfected with FLAG-tagged Btnl6 cDNA pMX-IRES-GFP served as a positive control. The predicted protein migrating under reducing conditions at the theoretical molecular weight of ~59 kDa for FLAG-tagged Btnl6 and ~58 kDa for non-tagged Btnl6 was detected with anti-FLAG antibody or Btnl6-specific polyclonal antibody. No bands were detected on gels immunoblotted with pre-immune serum. The β-actin immunoblot acts as a loading control. Data are representative of two experiments. GF: germ-free; CV: conventional.

In order to determine if the expression of Btnl1 and Btnl6 in the intestinal mucosa in the early postnatal period is dependent on the presence of intestinal microbiota, crude intestinal samples from adult mice maintained under strict GF conditions were analyzed with real-time PCR for the presence of *Btnl1* and *Btnl6* transcripts. As is evident in Fig. 2A, *Btnl1* and *Btnl6* RNA was readily expressed in GF animals and the level of RNA expression, although slightly lower, was comparable to RNA levels found in animals housed in CV conditions (Fig. 2A). To determine protein levels, small intestinal tissue derived from GF mice was immunostained with anti-Btnl1 antibody for Btnl1 detection, or used for western blot analysis for detection of Btnl6. Btnl1^+^ epithelial cells were detected in the intestine of GF animals with a frequency comparable to CV mice (Fig. 2B). Likewise, Btnl6 was present in lysates of small intestinal tissue of GF mice (Fig. 2C). Hence, these data show that Btnl1 and Btnl6 are expressed in the absence of intestinal microbiota and indicate that the delay in Btnl protein expression in the intestine of CV mice is not due to gut colonization.

### Expansion of Vγ7Vδ4 TCR IELs in the neonate gut associates with the appearance of the Btnl1 and Btnl6 proteins

**Figure 3:**
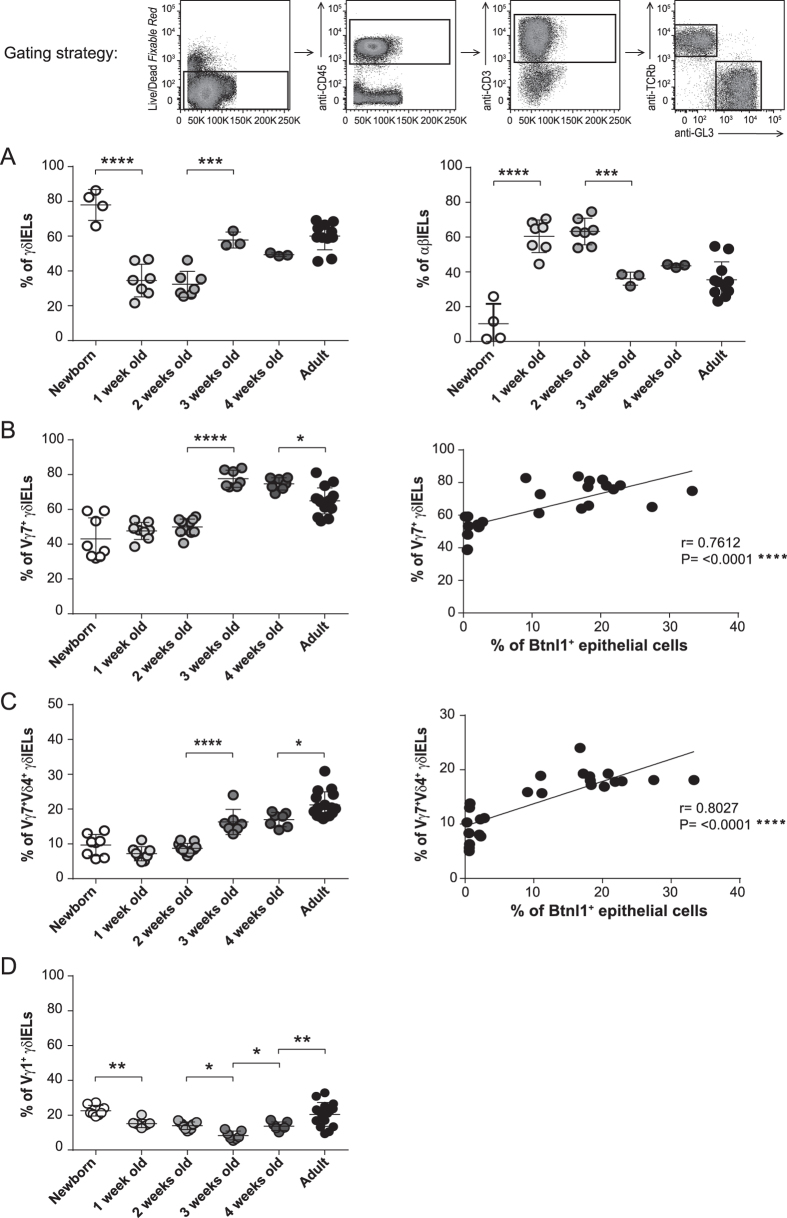
Btnl1 expression correlates with the presence of IELs bearing the Vγ7Vδ4 TCR. Small intestinal IELs from newborn (day 0), 1, 2, 3, and 4-week-old and adult C57BL/6 mice were analyzed for the expression of αβ and γδ TCR **(A)**, and the γδ TCR IELs for the expression of Vγ7, Vγ1, and Vδ4 chains **(B–D)**. 3–11 mice/group **(A)** and 7–16 mice/group **(B–D)** were analyzed and One-Way ANOVA followed by Holm-Sidak´s multiple comparisons test was used for statistical analysis. Correlation between Btnl1 expression and the percentage of Vγ7 TCR IELs **(B)** or Vγ7Vδ4 TCR IELs **(C)** during the mouse ontogeny was determined using the Pearson correlation test (*P ≤ 0.05, **P ≤ 0.01, ***P ≤ 0.001 and ****P ≤ 0.0001). Each dot in the correlation analysis in Fig. 3B,C represents % of Btnl1^+^ epithelial cells vs % of Vγ7 (Vδ4) TCR IELs of one mouse at a particular time-point. Newborn −8-week-old mice were used in this correlation analysis.

Our recent data show that Btnl1 promotes IEL proliferation and that Btnl1-Btnl6 heteromers increase the frequency of Vγ7Vδ4 TCR IELs in particular^6^. In view of the current results showing a delay in Btnl1 and Btnl6 protein expression in neonatal intestinal mucosa the relative frequencies of γδ and αβ TCR IELs and the relative frequencies of Vγ7 and Vγ1 chains, the principal chains utilized in C57BL/6 mice^24^, were determined. This analysis identified γδ TCR T cells as the predominant intraepithelial T cell subset comprising 80% (78% ± 9) of the total IELs at birth (Fig. 3A). The ratio between γδ:αβ IELs then dramatically inverted and at day 7, and until 2 weeks of age, αβ TCR T cells represented the dominant IEL subset (63% ± 8 αβ TCR IELs vs. 32% ± 7 γδ TCR IELs) (Fig. 3A). At the age of 3 weeks the frequency of γδ TCR T cells increased substantially (58% ± 5 γδ TCR IELs vs. 36% ± 4 αβ TCR IELs) and at this age the IEL composition was similar to the composition of IEL population in adult mice (Fig. 3A). The significant enrichment of γδ TCR T cells at week 3 coincided with the substantial increase in Btnl1 levels and the appearance of Btnl6 in the intestinal mucosa. To determine whether the appearance of the Btnl proteins coincided with increased Vγ7Vδ4 TCR levels, we examined the relative frequencies of Vγ7, Vδ4 and Vγ1 IELs. Our data show that within the γδ TCR IEL subset, cells expressing Vγ7 and cells expressing Vγ7Vδ4 TCR increased markedly between postnatal weeks 2 and 3 (from 50% ± 5 to 78% ± 5 and from 9% ± 1 to 16% ± 4, respectively) (Fig. 3B,C), and that the expansion of these cells correlated with a substantial increase in Btnl1 protein levels (Fig. 3B,C). In contrast, the ratio of Vγ1-bearing IELs was not specifically affected by the appearance of the Btnl proteins (Fig. 3D).

## Discussion

We have analyzed the ontogeny of Btnl1 and Btnl6 in the small intestine of neonatal mice. Our data identify a substantial delay in Btnl1 and Btnl6 protein expression in the neonatal intestinal epithelium and demonstrate that these proteins are absent in the small intestinal mucosa at birth and during the first 2–3 weeks of life. The delay in Btnl1 and Btnl6 protein expression was not reflected at the RNA level suggesting post-transcriptional mechanisms that regulate Btnl protein expression in the postnatal gut. Such mechanisms may involve microRNAs that function in RNA silencing and have the capacity to repress the translation of transcribed mRNAs^25^, or post-translational turnover of the protein. During early neonatal life, important changes occur in the intestine. The developing gut immune system is challenged by milk and microbial flora and later the diet of mice changes from milk to pelleted food leading to changes in microbial contents. This period is essential for a complete development of the mucosal immune system and we therefore assessed the impact of gut microbiota on Btnl expression by investigating the presence of Btnl1 and Btnl6 proteins in GF animals. We found that Btnl expression does not correlate with microbial exposure as mice housed in GF conditions had Btnl1 and Btnl6 protein levels which were not different from conventional mice. Although the presence of Btnl1 in 14-day-old pre-weaning pups kept mainly on a milk diet suggests that expression of Btnl proteins is not dependent on change of diet, we cannot determine whether Btnl expression is regulated by increasing exposure to dietary antigen before weaning, or if the expression is regulated by an unknown developmental factor.

We previously reported that Btnl1 can promote the expansion of small intestinal IELs, and that Btnl1 in a heteromeric protein complex with Btnl6, augments the expansion of γδ T cells bearing the Vγ7Vδ4 TCR in particular^6^. To examine if the appearance of the Btnl1 and Btnl6 proteins in the intestinal epithelium was associated with the expansion of γδ TCR cells we examined the percentage of Vγ7 and Vγ7Vδ4 bearing IELs in the neonatal mice. In agreement with previous reports investigating neonatal mouse small intestine^26^, we found that between 2 and 3 weeks of age γδ TCR IELs increased substantially, whereas the percentage of αβ TCR IELs decreased. Within the expanding γδ TCR IEL compartment Vγ7 and Vγ7Vδ4 TCR bearing T cells showed the greatest increase that correlated with the appearance of the Btnl proteins in the intestine. The expansion of γδ ΤCR IELs is indicative of a major influx of γδ TCR lymphocytes and/or a local expansion of resident γδ TCR IELs in the neonatal gut epithelium. Observations from rat IEL ontogeny in which relative IEL numbers were similar in normal and athymic neonates^27^, and from mouse studies in which γδ TCR IELs were regenerated in the small intestine in the absence of the thymus^28^, suggest that the IEL colonization process may be regulated by gut micro-environmental factors rather than by immigration of thymus-derived T cells. Intriguingly, representation of Vδ4-expressing IEL subsets is influenced by genes linked to the MHC Class II region^24^,^29^, which also contains the Btnl1 and Btnl6 genes. Although further experiments, for example using *Btnl*-/- approaches, will be necessary to confirm the association between Btnl1 and Btnl6 and the expansion of γδ TCR IELs, particularly those bearing the Vγ7Vδ4 TCR, these data support our recent *in vitro* results^6^ and further add strength to the evidence of a link between Btnl genes and the γδ expressing IEL repertoire in the intestinal intraepithelial compartment.

## Additional Information

**How to cite this article**: Lebrero-Fernández, C. and Bas-Forsberg, A. The ontogeny of Butyrophilin-like (Btnl) 1 and Btnl6 in murine small intestine. *Sci. Rep.* **6**, 31524; doi: 10.1038/srep31524 (2016).
